# Dataset of macrobenthic species in urban coastal reef environments in Brazilian northeast

**DOI:** 10.1016/j.dib.2020.105773

**Published:** 2020-06-02

**Authors:** Daniel Leite, Edson Vasconcelos, Pablo Riul, Natan Freitas, George Miranda

**Affiliations:** a Graduate Program in Development and the Environment, Federal University of Paraíba (Universidade Federal da Paraíba – UFPB), João Pessoa, Paraíba, Brazil; b Department of Health, Joaquim Nabuco University Center, Recife, Pernambuco, Brazil; c Department of Systematics and Ecology, Center of Exact and Natural Sciences, UFPB, João Pessoa, Paraíba, Brazil; d Graduate Program in Biological Sciences, UFPB, João Pessoa, Paraíba, Brazil

**Keywords:** Reef environments, Macrobenthic community, Occurrence frequency, Bioindication

## Abstract

This dataset presents the macrobenthic species occurrence frequency in four coastal intertidal reefs environment of Paraiba state Brazil. The species were classified in bioindicators groups based in morpho-anatomical, physiological and ecological characteristics. In the dry and wet season in each reef, the sampling units were randomly positioned and photographed inside a circular area with a 10 m radius. Thirty points were plotted over photos to quantify the percentage of species occurrence frequency. Taxons hard to identify by photo were grouped in a unique category. Currently, macrobenthic species are used as bioindicators of the local state of conservation by managing agencies. The population ecology monitoring of macrobenthic species with bioindicator potential is useful to recognize seasonal environmental patterns or local anthropic impactful. The research article on these data [1] will be published in the journal Ocean and Coastal Management for some interpretive insights. Title: Evaluation of the conservation status and monitoring proposal for the coastal reefs of Paraíba, Brazil: bioindication as an environmental management tool.

Specifications table

**Table:** 

Subject	Environmental Science
Specific subject area	Use of community data for bioindication of conservation status.
Type of data	Table
How data were acquired	To obtain the samples, we used a photographic camera (Nikon Coolpix W300 16 MP Zoom 35x) and built a polyvinyl chloride (PVC) pipe quadrat as the sampling unit. We analysed the photos with the program Coral Point Count with Excel extension. The sampling was conducted once a month during the dry (December 17, January 18, February 18) and rainy seasons (June 18, July 18 and August 18). The photos were taken on tides below 0.4 m, which occurred between 7 a.m. and 2 p.m. During these tides, the reefs were exposed for approximately 1 h
Data format	Raw Analyzed
Parameters for data collection	We consider only the intertidal zone of urban coastal reef environments, Paraíba, Brazil. Were excluded the wave breaking zone, sampling only the reefs central zone (>5 m from the reef edge).
Description of data collection	In each month at each reef, fifty 50 × 50 cm sampling units were randomly positioned inside a circular area with a 10 m radius. Each sampling unit was randomly placed an angle (every 15°) and a distance (with marks every 50 cm) from the center of the circle [1, 2]. Thirty random points were plotted in the images (samples) using the program Coral Point Count with Excel extensions (CPCe) to quantify the coverage percentage of the adopted taxonomic categories.
Data source location	City/Town/Region: João Pessoa and Cabedelo cities, Paraíba state. Country: Brazil. Latitude and longitude: 07°00′41″S; 34°48′58″W - Areia Vermelha Reef 6°59′04″S; 34°48′56″W – Formosa Reef 07°06′59’’S; 34°48′32’’W – Picãozinho Reef 07°09′02’’S; 34°47′14’’W – Seixas Reef DATUM: WGS 84
Data accessibility	The research article on these data was approved for publication in the journal Ocean and Coastal Management. Title: Evaluation of the conservation status and monitoring proposal for the coastal reefs of Paraíba, Brazil: bioindication as an environmental management tool.
Related research article	Authors: Leite, Daniel Silva Lula; Vasconcelos, Edson Regis Tavares Pessoa Pinho de; Riul, Pablo; Freitas, Natan Diego Alves de; Miranda, George Emmanuel Cavalcanti de. Title: Evaluation of the conservation status and monitoring proposal for the coastal reefs of Paraíba, Brazil: bioindication as an environmental management tool. Journal: Ocean and Coastal Management. In Press.

## Value of the data

• The dataset is useful for studies on community ecology in tropical reefs to a broad audience and, locally, it is a baseline for comparison with distinct reef conditions.
• The dataset is useful for public management agencies and managers of protected areas, being particularly interesting for the evaluation and monitoring of conservation status, which can easily be applicable in other reef environments in accordance with the classification system used.
• As this framework allows rapid assessment of reef conditions, it may stimulate the production of comparable information regarding the conservation status of these environments worldwide and it can be useful for the development of studies in marine macroecology.
• The data provides diagnosis of environmental quality, allowing better decision making by competent authorities and providing subsides for the management of these environments, aiming at the long-term maintenance of environmental quality and ecosystem services.

## 1 Data Description

Here we provide a dataset on a frequency occurrence of macrobenthic species in coastal reef environments in the Brazilian northeast and suggest a classification in different bioindicator species groups.

Multimedia Component 1 shows the absolute frequency of each species by sampling unit in the respective Month/Reef/Season. As is not possible to identify some species/genera using photographic methods we grouped these species in the same taxonomic operational unit.

Multimedia Component 2 shows the classification of species in different bioindicator groups using the classification criteria proposed by Orfanidis et al. (2011) [3]. The species classified in Group I are bioindicators of good environmental quality areas and the species from Group II are bioindicators of impacted areas.

## 2 Experimental design, materials, and methods

### 2.1 Study area

**Fig. 1:**
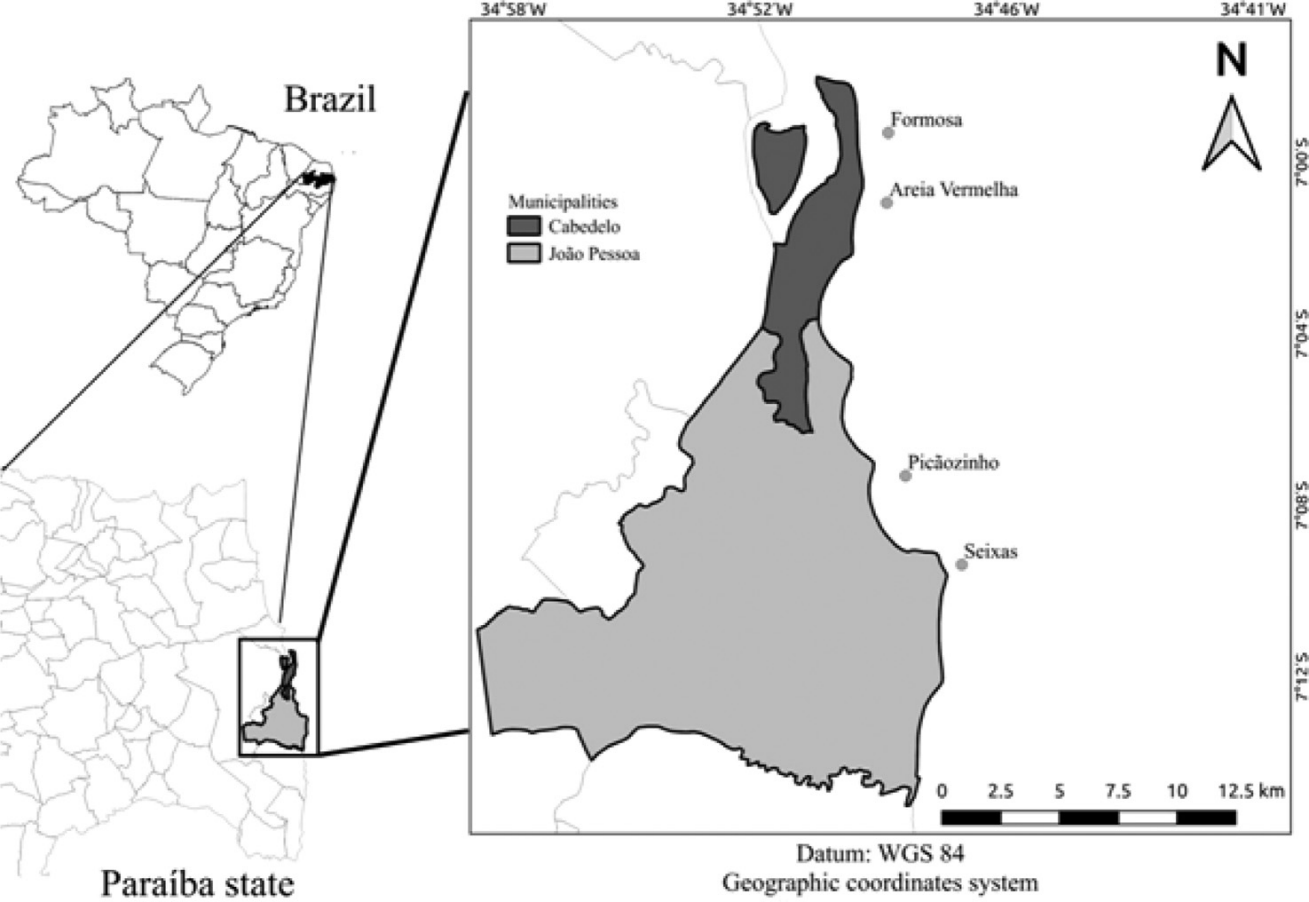
Map of the reef environments of Areia Vermelha, Formosa, Seixas and Picãozinho.

We sampled the intertidal zone of the reefs from in the central coastal strip of the Paraíba state (Fig. 1). These are (i) Formosa Reef and (ii) Areia Vermelha Reef in the municipality of Cabedelo and (iii) Picãozinho Reef (iv) and Seixas Reef in the municipality of João Pessoa. The reefs are exposed at tides below 0.4 m, with distance <1,5 km from the coast, based on banks of submerged sandstones and have a common geomorphological unit [4].

In the coast of Paraíba, the wet season starts in March and extends to August [5], with the peak occurring between April and July [6]. In September the dry season starts, ending in February [5], reaching its peak in October and November [6].

### 2.2 Sample processing and analysis

To quantify the percentage of species coverage, sampling was conducted once a month during the dry (December 17, January 18, February 18) and rainy seasons (June 18, July 18 and August 18). In each month at each reef, fifty 50 × 50 cm sampling units were randomly positioned inside a circular area with a 10 m radius. Each sampling unit was randomly placed an angle (every 15°) and a distance (with marcations every 50 cm) from the center of the circle [1,2]. Thirty random points were plotted in the images (samples) using the program Coral Point Count with Excel extensions (CPCe) to quantify the% coverage of the adopted taxonomic categories.

The wave breaking zone was avoided, and the centers of the reefs (at least 5 m from the reef edge) were sampled. Taxa that were difficult to identify using the photographic method were grouped into a single category. The taxonomic classification and adopted update were proposed by Guiry and Guiry [7] and the WoRMS Editorial Board [8].

To classify the species in different groups of bioindicators, we used the classification criteria proposed by Orfanidis et al. (2011) [3], accordingly to species morpho-anatomical, physiological and ecological characteristics. Although the classification is for macrophytes in rocky shore environment and transitional waters, we understand that these criteria are valid to extend to any macrobenthic species in any kind of ecosystem. For the classification of zoanthid species, was excluded the criteria “thallus morphology”.

The species were classified in Group I and Group II. Species classified in Group I are bioindicators of good environmental quality, while species in Group II are bioindicators of impacted areas. Each group was divided into sub-groups: Group I - IA, IB and IC, the species classified as IA are bioindicator of areas with higher-level of environmental quality, followed by IB and IC, respectively; Group II - IIA and IIB, the species classified as IIB are bioindicator of higher impactful areas when compared with IIA. The classification of species was based on works realized on environmental coastal reefs from the Atlantic Ocean, available in Multimedia Component 2.

## Declaration of Competing Interest

The authors declare that they have no known competing financial interests or personal relationships which have, or could be perceived to have, influenced the work reported in this article.

## Appendix Supplementary materials

Image, application 1

Image, application 2
